# Neonatal tactile stimulation at birth in a low-resource setting

**DOI:** 10.1186/s12887-018-1279-4

**Published:** 2018-09-20

**Authors:** Andrea Pietravalle, Francesco Cavallin, Anna Opocher, Stefania Madella, Maria Elena Cavicchiolo, Damiano Pizzol, Giovanni Putoto, Daniele Trevisanuto

**Affiliations:** 1 Department of Woman’s and Child’s Health, University of Padua, Azienda Ospedaliera di Padova, Via Giustiniani 3, 35128 Padua, Italy; 2 Independent statistician, Solagna, Italy; 3 Doctors with Africa CUAMM, Padua, Italy

**Keywords:** Delivery room, Low-resource setting, Neonatal resuscitation, Newborn, Stimulation

## Abstract

**Background:**

Stimulation is the most common intervention during neonatal resuscitation at birth, but scarce information is available on the actual methods, timing and efficacy of this basic step. To evaluate the occurrence, patterns and response to tactile stimulation at birth in a low-resource setting.

**Methods:**

We reviewed 150 video recordings of neonatal resuscitation at Beira Central Hospital (Beira, Mozambique). Timing, method, duration and response to tactile stimulation were evaluated.

**Results:**

One hundred two out of 150 neonates (68.0%) received stimulation, while the remaining 48 (32.0%) received positive pressure ventilation and/or chest compressions directly. Overall, 546 stimulation episodes (median 4 episodes per subject, IQR 2–7) were performed. Median time to the first stimulation episode was 134 s (IQR 53–251); 29 neonates (28.4%) received stimulation within the first minute after birth. Multiple techniques of stimulation were administered in 66 neonates (64.7%), while recommended techniques (rubbing the back or flicking the soles of the feet) only in 9 (8.8%). Median duration of stimulation was 17 s (IQR 9–33). Only 9 neonates (8.8%) responded to stimulation.

**Conclusions:**

In a low-resource setting, stimulation of newly born infants at birth is underperformed. Adherence to international guidelines is low, resulting in delayed initiation, inadequate technique, prolonged duration and low response to stimulation. Back rubs may provide some benefits, but large prospective studies comparing different methods of stimulation are required.

## Background

Initiation of breathing is critical in the physiologic transition from intra-uterine to extra-uterine life [1]. In high-resource settings, approximately 85% of babies born at term initiate spontaneous respirations within 10 to 30 s after birth, 5–10% respond to simple stimulation, 3–6% start breathing after basic resuscitation (positive-pressure ventilation, PPV) and less than 1% require advanced resuscitation (intubation, chest compressions and drugs) [2]. Resuscitation includes different interventions based on progressive steps (Table 1). In low-resource settings, a large observational study in a rural hospital in Tanzania suggested that 85% of infants would require only simple newborn care, whereas 15% would need stimulation, including 7% requiring bag-mask ventilation and less than 1% requiring advanced care [3].

**Table 1:** Steps of neonatal resuscitation and interventions

Initial steps	Ventilation	Chest compressions	Medications
Warming Suctioning Stimulation Evaluation	Face-mask ventilation Laryngeal-mask ventilation^a^ Intubation^a^	Chest compressions with two-thumb techniques	Adrenaline^a^ Volume expanders^a^

^a^ interventions not available in the study setting

The need for neonatal resuscitation is most urgent in low-resource settings, where access to intrapartum obstetric care is poor and long-term impairments from intrapartum-related events represent a heavy burden [4]. While babies requiring advanced resuscitation may not survive without ongoing ventilation and neonatal intensive care, neonatal mortality from intrapartum-related events in low- and middle-resource settings can be reduced by 30% with basic training in neonatal resuscitation [5]. Expert consensus estimates a 10% reduction in intrapartum-related deaths with immediate newborn assessment and stimulation alone [6].

Although stimulation is the most common intervention during neonatal resuscitation/stabilization at birth and is also recommended by all neonatal resuscitation guidelines, [7–9] scarce information is available on the actual methods, timing and efficacy of this basic step. A limited number of retrospective observational studies in high-resource settings have investigated this topic so far. Dekker et al. reviewed 164 neonatal stimulations at birth of infants with a gestational age of < 32 weeks and reported large variability in the use of tactile stimulation without a clearly demonstrable effect on infants [10]. Gaertner et al. evaluated video recordings of 75 stimulated infants, including very preterm infants, and suggested that truncal stimulation (drying, chest rubs and back rubs) might be more effective than foot flicks [11]. All authors indicated the need for further studies in order to confirm such preliminary findings. It is noteworthy that these results might underestimate the number of stimulations received by healthy near-term and at term newborns. Moreover, the number and types of stimulation may vary in different settings or with less experienced staff.

The aim of this study was to evaluate the occurrence, patterns and response to tactile stimulation at birth in newly born infants in a low-resource setting.

## Methods

### Setting

This study was performed at Beira Central Hospital (Beira, Mozambique) where about 4500 deliveries occur every year. Beira Central Hospital is the referral hospital for a geographical area that covers about 7 million people, with large referral services for maternal and neonatal care in the province [12].

### Study design

This study presents a secondary analysis of data collected during a prospective study on education in neonatal resuscitation using videorecording. The main study was designed to assess the impact of a Neonatal Resuscitation Program course followed by a continuous refresher training on clinical practice of midwives at Beira Central Hospital [13]. The research protocol was approved by the National Committee of Bioethics (Ref. 315/CNBS/13; November, 1, 2013) and by the Minister of Health of the Republic of Mozambique (Ref. 08/GMS/002/2014; January, 7, 2014). Parental consent to record neonatal delivery room management and to use the data was obtained before every delivery. Written informed consent was given by parents and caregivers for clinical records to be used in this study. All information, including informed consent and all the material used in the study was written in Portuguese in a clearly understandable form.

### Patients

All 150 neonates who were enrolled in the original study were considered for inclusion in the present analysis. All of them needed resuscitation of some form at birth. Neonates who required stimulation were included in the analysis. Resuscitation was defined as any intervention performed by healthcare providers: initial steps (drying and stimulation), bag mask ventilation, and/or chest compressions. Lack of parental consent was the only exclusion criterion.

### Procedures

Neonatal resuscitation was performed routinely under radiant warmers in the delivery room or in the obstetric operating room and was based on an adapted algorithm of 2010 American Heart Association Guidelines, with the exclusion of intubation and medication administration [13, 14].

Stimulation was defined as any intervention provided to the baby under the infant warmer, [6, 11, 15] including back rub (any rub to the back), foot flick (any stimulation targeting the sole, i.e. flicking or rubbing), chest rub (any rub to the front or side of the thorax) and abdomen rub (any rub to the front or side of the abdomen). A stimulation was recorded as a separate episode if there was a gap of at least 2 s between two stimulations or if the nature of the stimulation changed. Concurrent stimulations (i.e. flicking the foot while rubbing the chest) were recorded as separate stimulations [11]. Only the stimulations that led to a complete newborn recovery, without need for further resuscitation, were considered effective.

All interventions at birth were video-recorded with a camera installed above the radiant warmers and data were collected until the end of resuscitation maneuvers or until the video was stopped because the infant transitioned well and was brought to the mother. Two researchers (DT and AP) drafted a categorical scheme based on Gaertner et al. [11] to identify the patterns of stimulation objectively. Two researchers (AP and AO) reviewed and evaluated all 150 videos of neonatal resuscitation, with a third researcher (SM) resolving any conflicts. Time of birth was defined as the time the Apgar clock was started or birth was announced [11].

### Video recording

Interventions were recorded using a webcam for video monitoring (ENXDVR-4C, Encore Electronics. www.encore-usa.com), consisting of 1 fixed camera installed above the radiant warmers both in the delivery room and in the operating room. The cameras provided a 24-h video recording without audio. The image was zoomed to show only the neonate and the hands of the resuscitation team. Parents, obstetric procedures and faces of the caregivers were not visible [16]. The video camera displayed a continuous time readout at the bottom of the recorded image allowing timing of performed procedures to the nearest second. All videos were stored on a hard disk and sent to the coordinating center (University of Padua). In order to protect the identities of the subjects and the data, all data about resuscitation date and location were removed, and shipment was insured. All 150 recordings were complete and of good quality.

### Outcomes

The main variable of interest was the response to stimulation defined as the complete newborn recovery, i.e. spontaneous breathing without need for PPV. The initiation time, the duration and the technique of stimulation were also evaluated.

#### Statistical analysis

This study presents a secondary analysis of data collected during a prospective study on education in neonatal resuscitation using videorecording. Thus, a convenience sample consisting of all 150 videos of the original study was analyzed. Continuous data were summarized using median and interquartile range (IQR), and categorical data as number and percentage. Data were compared between two groups using Mann-Whitney test (continuous data) or Fisher’s exact test (categorical data). Correlation between continuous variables were evaluated using Spearman rank correlation coefficient. All test were 2-sided and a *p*-value less than 0.05 was considered statistically significant. Statistical analysis was performed using R 3.3.0 (R Foundation for Statistical Computing, Vienna, Austria) [17].

## Results

**Fig. 1:**
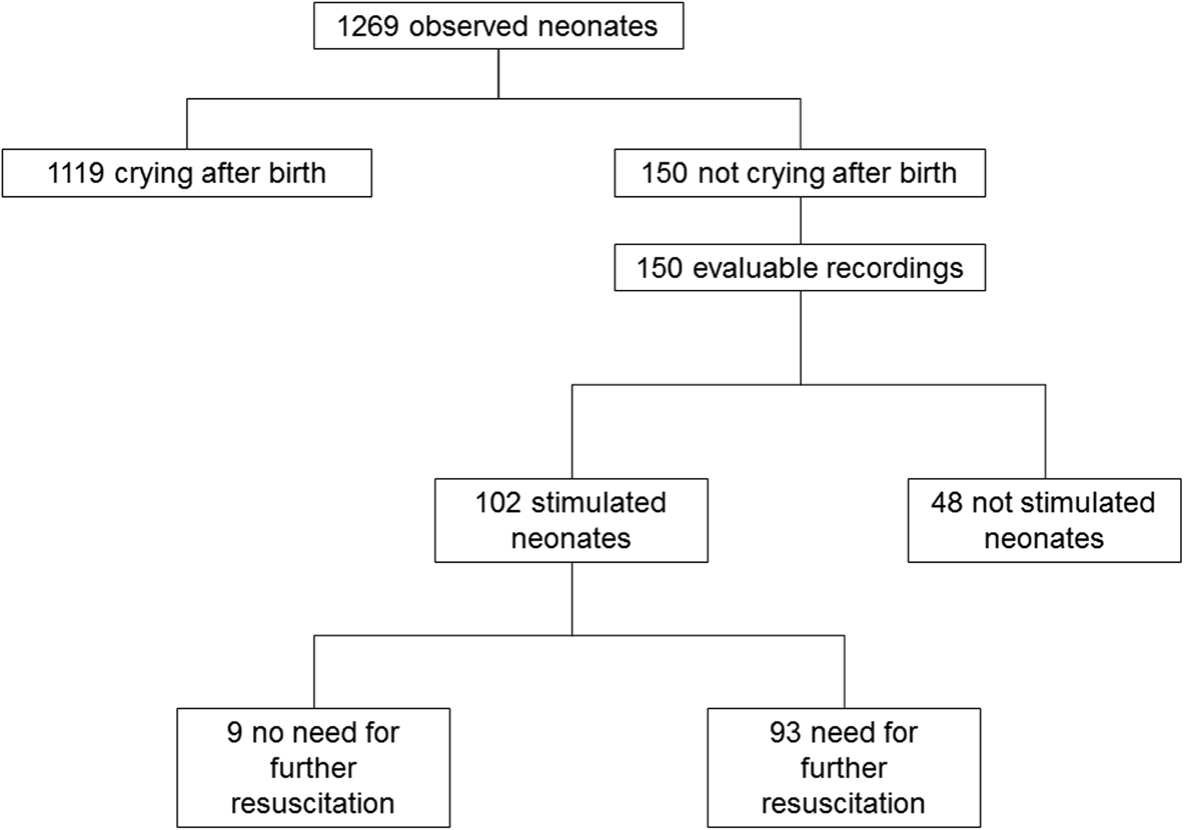
Flowchart of included neonates

Of the 150 video recordings, 102 neonates (68.0%) received stimulation because of apnea (4 neonates), hypotonia (12 neonates) or both (86 neonates). The remaining 48 neonates went directly.

to PPV and/or chest compressions and were excluded from the analysis (Fig. 1). Characteristics of included neonates are shown in Table 2. Median gestational age was 38 weeks (IQR 37–40) and median birth weight was 2875 g (IQR 2200–3280).

**Table 2:** Demographics

No. of stimulated neonates	102
Reason for stimulation	
Apnea	4 (3.9)
Hypotonia	12 (11.8)
Apnea and hypotonia	86 (84.3)
Mode of delivery	
Vaginal	53 (52.0)
Caesarean	49 (48.0)
Sex	
Male	66 (64.7)
Female	36 (35.3)
Birth weight, grams	2875 (2200–3280)
Gestational age, weeks	38 (37–40)
Apgar score at 1 min	5 (3–6)
Apgar score at 5 min	6 (4–7)

Data expressed as No. (%) or median (IQR)

**Fig. 2:**
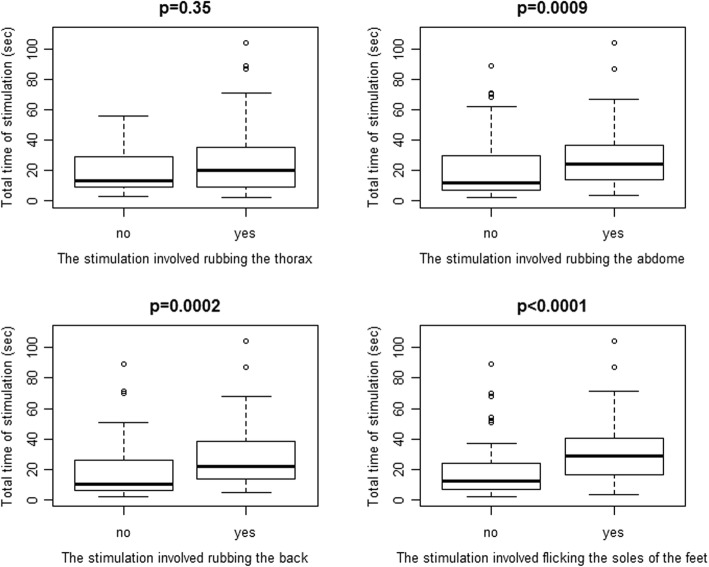
Total time of stimulation

**Fig. 3:**
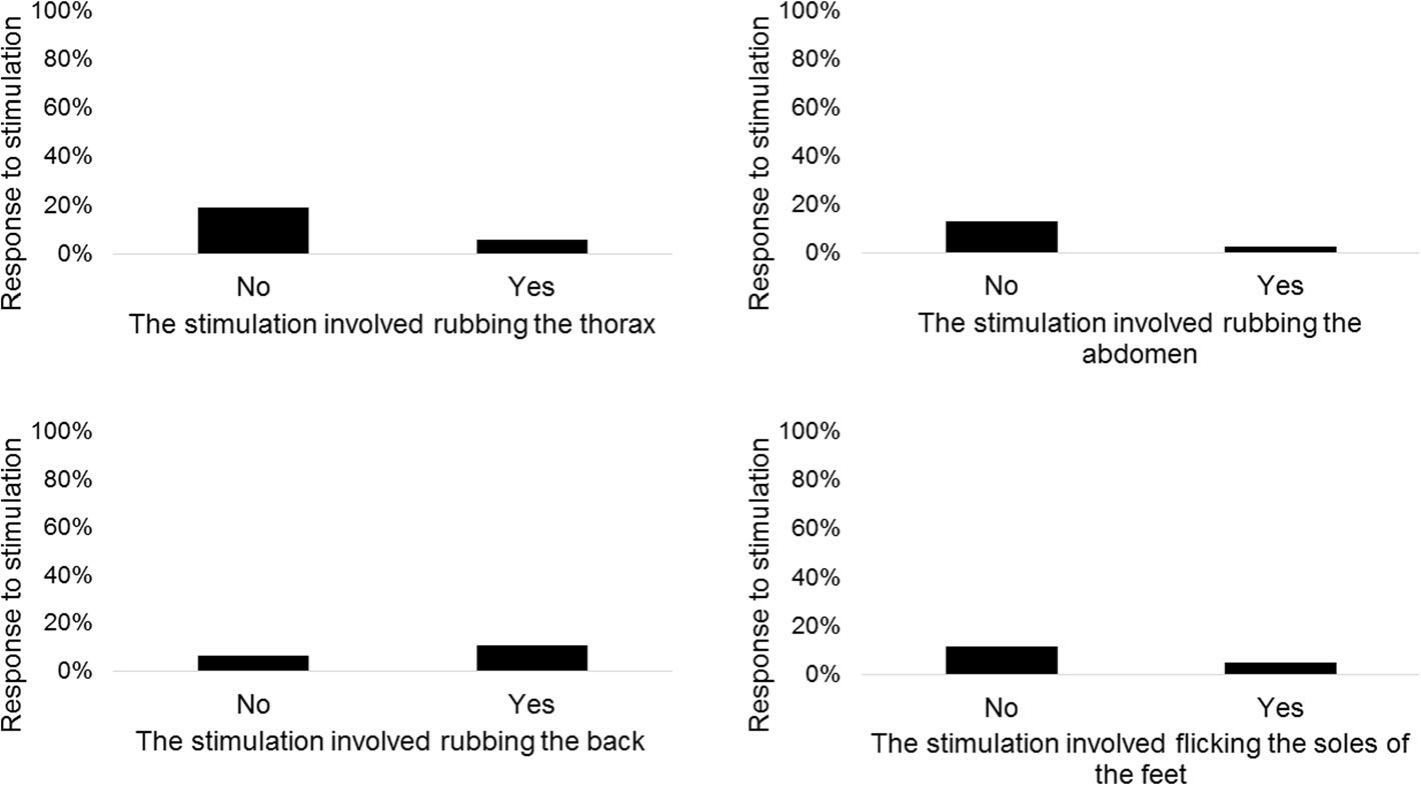
Response to stimulation

Overall, 546 stimulation episodes (median 4 episodes per subject, IQR 2–7) were performed. Description of stimulations is reported in Table 3. Median time elapsed from birth to the first stimulation was 134 s (IQR 53–251); 29 neonates (28.4%) received stimulation within the first minute after birth. Figure 2 shows the total time of stimulation comparing when a specific procedure was performed or not. Rubbing the thorax (upper left) was not associated with total time of stimulation (*p* = 0.35). Instead, rubbing the abdomen (upper right, *p* = 0.0009), rubbing the back (lower left, *p* = 0.0002) and flicking the soles of the feet (lower right, *p* < 0.0001) were associated with longer total time of stimulation compared to not performing the corresponding procedure (Fig. 2). The number of different techniques included in the stimulation was associated with longer total time of stimulation (Spearman rho 0.57, p < 0.0001), but not with time to first stimulation episode (Spearman rho 0.05, *p* = 0.62). Nine neonates (8.8%) responded to stimulation. The low number of responding neonates prevented any meaningful analyses, but data suggested that rubbing the back might increase the efficacy of the stimulation (Fig. 3).

**Table 3:** Stimulations

Timing and number of stimulations	Time elapsed from birth to stimulation, seconds	134 (53–251)
	Duration of the first stimulation episode, seconds	4 (2–7)
	Number of stimulations/neonate	4 (2–7)
	Total time of stimulation, seconds	17 (9–33)
Technique of stimulation	The stimulation involved rubbing the thorax	81 (79.4)
	The stimulation involved rubbing the abdomen	40 (39.2)
	The stimulation involved rubbing the back	55 (53.9)
	The stimulation involved flicking the soles of the feet	40 (39.2)
	Truncal stimulation (rubbing the thorax and/or the back)	98 (96.1)
	Technique of stimulation	
	Single technique	36 (35.3)
	Two techniques	31 (30.4)
	Three techniques	22 (21.6)
	Four techniques	13 (12.7)

Data expressed as No. (%) or median (IQR)

Single technique: 26 only thorax, 1 only abdomen, 6 only back, 3 only feet

## Discussion

The present study evaluated the methods, timing and response to tactile stimulation in late preterm and full-term infants in low-resource settings. To our knowledge, only two retrospective studies conducted in preterm infants in high-resource settings, investigated such aspects [10, 11]. In these studies, the effect of stimulation was assessed as recovery of heart rate > 100 bpm and/or regaining breathing/increased breathing effort, [10] or as changes in crying, movement and grimace. [11] In the present study, the stimulation was considered as effective when it provided a complete newborn recovery, avoiding the need for PPV.

Our main result was the very low number of infants (9%) who responded to stimulation. A previous study in Tanzania suggested that around 50% of newly born infants might respond to stimulation thus avoiding the need for PPV [3]. This difference could be related to some study features such as the different definition of response to stimulation, the inclusion of infants needing resuscitation under the infant warmer in our series and the resulting longer delay of initiation of stimulation. Face-mask ventilation is a crucial step in neonatal resuscitation but it is a difficult skill to teach and maintain in low-resource settings [18]. Therefore, effective stimulation during the first steps of resuscitation may reduce the need for additional neonatal resuscitation procedures such as face-mask ventilation or intubation. Expert consensus indicates that immediate newborn assessment and stimulation alone may avoid 1 out of 10 intrapartum-related deaths [6].

In our series, the recommended stimulation techniques (i.e. rubbing the back or flicking the soles of the feet [19]) were rarely performed alone and were usually associated with others techniques (i.e. rubbing the abdomen or the thorax), thus preventing any meaningful conclusions on efficacy. However, stimulations including rubbing the back seemed to be promising in terms of response rate, but the low number of responding infants suggested caution in interpreting the observed results. Low response rate and large variation in the use of tactile stimulation were also reported in the two studies in high-resource settings. Dekker et al. observed that 80% of their study cohort received recommended stimulation technique and 18% of stimulation episodes were effective, while the overall effect per infant remained unclear. [10] Gaertner et al. suggested that truncal stimulation (i.e. drying, chest rubs and back rubs) might increase the response to stimulation, but the low sample size prevented definitive conclusions [11].

It is noteworthy that 1 out of 3 infants did not receive stimulation in our series, thus confirming available data in high-resource settings [10, 11]. While the number of immature infants and medical team’s focus on respiratory support might have been the reasons for skipping stimulation in other studies, [10, 11] we believe that low-resource setting and less-experienced staff were more likely to be associated with skipping stimulation in our series. In fact, a previous study showed limited ability of the staff to adhere to the resuscitation algorithm [18].

Overall, our data showed low adherence to the international guidelines in term of initiation, duration and method of stimulation [7–10]. The initiation of stimulation was frequently delayed, with only 28.4% of infants receiving stimulation within the first minute after birth. In high-resource settings, Dekker et al. also reported delayed initiation of stimulation (less than 25% of infants receiving stimulation within the first minute after birth), [10] while Gaertner et al. reported prompt initiation (median time to first stimulation of 19 s) [11]. The method of stimulation was mostly inadequate, with 35% of infants stimulated using a single technique and only 9% as recommended (i.e. rubbing the back or flicking the soles of the feet). The majority of infants were stimulated using multiple techniques, in agreement with findings in high-resource settings [10, 11]. As consequence, the duration of stimulation was longer than recommended, as reported also by Dekker et al. [10] The prolonged duration of stimulation represents an additional hazard for infants, because it delays the initiation of PPV thus compromising the efficacy of the overall resuscitation process. Therefore, the low adherence to the international guidelines might have contributed to the low response to stimulation in our series.

The strengths of the present study include the evaluation of the response to stimulation as complete newborn recovery preventing the need for PPV, the objective evaluation of resuscitation procedure by using video-recording and the detailed review of stimulation maneuvers.

This study has also some limitations. First, it is a secondary analysis of data collected during a previous prospective study [13]. The original study was designed to video record only the resuscitation maneuvers applied when the newborns were moved under the infant warmer, thus the analysis might not include some depressed newborns who recovered after stimulation administered immediately after birth. Second, the low number of responding neonates and the heterogeneity of combinations of stimulation techniques did not provide strong indications on efficacy of single stimulation approaches.

## Conclusions

In low-resource settings, stimulation of newly born infants needing resuscitation is underperformed. Adherence to international guidelines is low, resulting in delayed initiation, inadequate technique, prolonged duration and low response to stimulation. Back rubs may provide some benefits, but large prospective studies comparing different methods of stimulation are required.

## Abbreviations

IQR: Interquartile range
PPV: Positive-pressure ventilation
